# Expression of genes for Kisspeptin (*KISS1*), Neurokinin B (*TAC3*), Prodynorphin (*PDYN*), and gonadotropin inhibitory hormone (*RFRP*) across natural puberty in ewes

**DOI:** 10.14814/phy2.14399

**Published:** 2020-03-13

**Authors:** Qun Li, Jeremy T. Smith, Belinda Henry, Alexandra Rao, Alda Pereira, Iain J. Clarke

**Affiliations:** ^1^ Department of Physiology Neuroscience Program Monash Biomedicine Discovery Institute Monash University Melbourne VIC Australia; ^2^ Present address: School of Anatomy, Physiology & Human Biology The University of Western Australia Nedlands WA USA; ^3^ Present address: Faculty of Veterinary and Agricultural Sciences The University of Melbourne Parkville VIC USA

**Keywords:** gonadotropin releasing hormone, gonadotropins, hypothalamus

## Abstract

Expression of particular genes in hypothami of ewes was measured across the natural pubertal transition by in situ hybridization. The ewes were allocated to three groups (*n* = 4); prepubertal, postpubertal and postpubertally gonadectomized (GDX). Prepubertal sheep were euthanized at 20 weeks of age and postpubertal animals at 32 weeks. GDX sheep were also euthanized at 32 weeks, 1 week after surgery. Expression of *KISS1*, *TAC3,* *PDYN* in the arcuate nucleus (ARC), *RFRP* in the dorsomedial hypothalamus and *GNRH1* in the preoptic area was quantified on a cellular basis. *KISS1R* expression by *GNRH1* cells was quantified by double‐label in situ hybridization. Across puberty, detectable *KISS1* cell number increased in the caudal ARC and whilst *PDYN* cell numbers were low, numbers increased in the rostral ARC. *TAC3* expression did not change but *RFRP* expression/cell was reduced across puberty. There was no change across puberty in the number of *GNRH1* cells that expressed the kisspeptin receptor (*KISS1R*). GDX shortly after puberty did not increase expression of any of the genes of interest. We conclude that *KISS1* expression in the ARC increases during puberty in ewes and this may be a causative factor in the pubertal activation of the reproductive axis. A reduction in expression of *RFRP* may be a factor in the onset of puberty, removing negative tone on GNRH1 cells. The lack of changes in expression of genes following GDX suggest that the effects of gonadal hormones may differ in young and mature animals.

## Graphical abstract

Kisspeptin is a major regulator of GnRH secretion and its function has been shown to be mandatory for pubertal transition. In this article, we examined the expression of *KISS1*, *TAC3*, *PDYN*, *RFRP*, *GNRH1* and *KISS1R* in specific areas of hypothalamus across puberty in ewe. We conclude that *KISS1* expression in the ARC increases during puberty in ewes and this may be a causative factor in the pubertal activation of the reproductive axis and a reduction in expression of *RFRP* may be a factor in the onset of puberty, removing negative tone on GNRH1 cells.

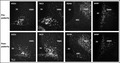

## 1 INTRODUCTION

Puberty is typified by an increase in the secretion of gonadotropin releasing hormone (GnRH) which drives an increase in gonadotropins secretion from the pituitary gland (Clarke & Pompolo, 2005; Ojeda, Roth, et al., 2006), leading to activation of the gonads. GnRH neurons are controlled by a number of interactive neuronal pathways which are regulated by internal signals and external cues (Clarke, 2015; Clarke & Arbabi, 2015; Clarke, Campbell, Smith, Prevot, & Wray, 2011; Ojeda, Lomniczi, et al., 2006; Tena‐Sempere, 2012; Terasawa & Fernandez, 2001), many of which may be involved in the process of puberty. Although much is known about neuronal systems that regulate GnRH secretion, the neurochemical basis of integrated control of puberty remains only partially understood. In higher primates, there is a “brake” on the secretion of GnRH and gonadotropins prior to puberty that is not due to feedback effects of steroids from the gonads (Plant, 2015; Plant & Shahab, 2002). In species such as the sheep, however, pulsatile GnRH secretion and gonadotropin secretion is apparent in the prepubertal period (I'Anson et al., 2000) but is held in check by an enhanced negative feedback action of estrogen (Foster & Ryan, 1979).

Since realization that kisspeptin is a major regulator of GnRH secretion (de Roux et al., 2003; Gottsch et al., 2004; Irwig et al., 2004; Messager et al., 2005; Seminara et al., 2003), its function was shown to be mandatory for pubertal transition (de Roux et al., 2003; Seminara et al., 2003). Nevertheless, the question as to whether altered function of kisspeptin is the primary and essential driver of the increase in GnRH that occurs at the time of puberty remains a matter of debate. There are two populations of kisspeptin neurons in hypothalamus which are differentially regulated by sex steroids. One population in the anteroventral periventricular nucleus (AVPV) in rodent or preoptic area (POA) in species such as sheep is involved in the positive feedback action of estrogen that causes the GnRH/luteinizing hormone (LH) surge in females (Hoffman, Le, Franceschini, Caraty, & Advis, 2011; Robertson, Clifton, Iglesia, Steiner, & Kauffman, 2009; Smith, Li, Pereira, & Clarke, 2009; Smith, Popa, Clifton, Hoffman, & Steiner, 2006). A second group of kisspeptin cells is located in the arcuate nucleus (ARC) and relays the negative feedback effects of sex steroid on GnRH secretion in both sexes (Franceschini et al., 2006; Smith, Cunningham, Rissman, Clifton, & Steiner, 2005). In sheep, kisspeptin neurons in caudal ARC also initiate the positive feedback effect of estrogen on GnRH secretion (Estrada, Clay, Pompolo, Smith, & Clarke, 2006; Smith, 2008; Smith et al., 2009), which is potentiated by activation of the preoptic kisspeptin neurons at the time of the surge (Hoffman et al., 2011).

Mutations in either the kisspeptin gene (*KISS1*) or the kisspeptin receptor (*KISS1R*) in humans and gene knockout in mice causes pubertal failure (d'Anglemont de Tassigny & Colledge, 2010; Dungan Lemko & Elias, 2012; de Roux et al., 2003; Seminara et al., 2003; Topaloglu et al., 2012). Nevertheless, a range of other gene mutations can also cause failure of puberty (Silveira, Trarbach, & Latronico, 2010). Developmental changes in *KISS1* expression have been described in many species, with variable patterns within and between species. In mice and rats, *KISS1* is expressed in the ARC before birth but some studies did not detect significant changes in expression in the ARC at the time of puberty in either species (Gill et al., 2010; Han et al., 2005; Navarro et al., 2012). Others (Molnar et al., 2016) have reported an increase in expression of *KISS1* in male mice at the time of puberty. Increased *KISS1* expression in the early stages of puberty has been observed in both male and female monkeys and rats (Bentsen et al., 2010; Shahab et al., 2005; Takase et al., 2009). No change in *KISS1* expression was observed in the ARC of female pigs across puberty (Ieda et al., 2014).

In female sheep, the enhanced negative feedback of estradiol‐17β (E2) in the prepubertal period is lost at puberty onset (Foster & Ryan, 1979). One study showed that, in ovariectomized (OVX)‐E2‐treated ewes, escape from the estrogenic “clamp” occurs at 35 weeks, typified by a marked increase in pulsatile LH secretion (Redmond et al., 2011). There was, however, no change in *KISS1* expression in the ARC of these animals, although an increase was observed in the POA by 30 weeks. In another study, immunohistochemistry showed the presence of more kisspeptin cells in ARC in adult ewes (>9 month) than in prepubertal ewes (5–6 months) (Nestor et al., 2012), consistent with the notion that kisspeptin may facilitate if not initiate puberty. No studies have been performed on gonad‐intact ewes across the pubertal transition to determine any potential change in *KISS1* gene expression.

Kisspeptin cells of the ARC also express *PDYN* and *TAC3*, the genes encoding dynorphin (DYN) and neurokinin B (NKB) (Goodman et al., 2007), leading to nomenclature of “KNDY” cells (Lehman, Coolen, & Goodman, 2010; Maeda et al., 2010; Navarro et al., 2009; Rance, Krajewski, Smith, Cholanian, & Dacks, 2010). NKB may act in an “autocrine” manner to stimulate kisspeptin secretion, whereas DYN inhibits KNDY cell function (Goodman, Coolen, & Lehman, 2014; Grachev et al., 2014; Sakamoto et al., 2012). Mutations in *TAC3* or the NKB receptor cause hypogonadotropic hypogonadism in humans (Gianetti et al., 2010; Topaloglu et al., 2009). NKB agonists can stimulate LH secretion prior to puberty in rats (Ruiz‐Pino et al., 2012), ewes (Nestor et al., 2012) and juvenile primates (Ramaswamy et al., 2010). In rats, developmental changes of *TAC3* expression show a steady increase from birth until the juvenile stage, but there is little change in the transition through puberty (Navarro et al., 2012). Most recently, it has been proposed that NKB/kisspeptin interaction is enhanced during the pubertal transition in female, but not male rhesus monkeys (Garcia, Keen, Kenealy, Seminara, & Terasawa, 2018). Immunoreactive NKB cell numbers were seen to be similar in prepubertal (5–6 months) and postpubertal ewes (>9 months) (Nestor et al., 2012).

DYN has an inhibitory effect on GnRH neurons as do other opioid peptides (Goodman et al., 2004; Navarro et al., 2009; Yen, Quigley, Reid, Ropert, & Cetel, 1985). This may be due to its action on kisspeptin cells or directly upon GnRH cells, which express the relevant receptor (Weems et al., 2016). In sheep, DYN neurons in the ARC appear to mediate the negative feedback effect of progesterone on GnRH/LH pulse secretion (Goodman et al., 2004, 2011; Goodman, Parfitt, Evans, Dahl, & Karsch, 1995). Thus, the numbers of DYN neurons are reduced in OVX ewes and restored by progesterone treatment (Foradori, Goodman, Adams, Valent, & Lehman, 2005). This is consistent withresults in human studies (Rometo & Rance, 2008), but not with data on manipulation by sex steroids in rats and nonhuman primates (Eghlidi, Haley, Noriega, Kohama, & Urbanski, 2010; Navarro et al., 2009). There are few studies of the role that DYN might play during puberty, but administration of a kappa receptor antagonist did advance puberty in female rats (Nakahara et al., 2013).

Gonadotropin inhibitory hormone (GnIH), also known as RF‐amide‐related peptide (RFRP) is a negative regulator of GnRH/gonadotropin function in various species, including sheep (Clarke et al., 2008, 2011, 2012; Clarke & Parkington, 2014; Clarke & Smith, 2010; Gibson et al., 2008; Johnson, Tsutsui, & Fraley, 2007; Kadokawa et al., 2009; Kriegsfeld et al., 2006; Murakami et al., 2008; Tsutsui et al., 2000). Administration of GnIH antisense oligonucleotide to juvenile male rats increased plasma LH levels and testicular weight (Johnson & Fraley, 2008) and RFRP receptor knock‐out mice have elevated plasma LH levels (Leon et al., 2014). In both studies, animals were fertile and had normal pubertal onset, providing no strong evidence that a change in GnIH function is important in reproductive development. On the other hand, *RFRP* expression changes with development in rodents, with a postnatal rise, a peak in expression at the time of puberty, and a decline in adulthood in both rats and mice (Iwasa et al., 2012; Poling, Kim, Dhamija, & Kauffman, 2012; Quennell, Rizwan, Relf, & Anderson, 2010). Strangely, RFRP‐3 stimulates LH secretion in male mice but is inhibitory in females (Ancel, Inglis, & Anderson, 2017). How this relates to control of the onset of puberty in either sex is not clear.

This study aimed to clarify the role of kisspeptin, NKB, DYN in the ARC, and RFRP in the DMH across puberty in ewes by measuring expression of the genes for the peptides. In addition, we examined if removal of sex steroid feedback by GDX shortly after puberty indicated whether the feedback loops evident in adult animals are fully developed at this time.

## 2 MATERIAL AND METHODS

All sheep were maintained at the Monash University Sheep Facility (Werribee) and the experiments were carried out in accordance with the Code of Practice for the Care and Use of Animals for Experimental Purposes provided by the National Health and Medical Research Council/Commonwealth Scientific and Industrial Research Organisation/Australian Animal Commission. The work was approved by the Monash University, School of Biomedical Sciences Animal Ethics Committee.

### 2.1 Animals and experimental design

Corriedale ewes were born in September in the Southern Hemisphere and were allocated into three groups (*n* = 4), being prepubertal, postpubertal and postpubertally gonadectomized (GDX). Prepubertal sheep were euthanized at 20 weeks (February). The estrous cycles of postpubertal intact ewes were synchronized by an injection (i.m.) of a synthetic prostaglandin (Estrumate, 125 µg; Pitman‐Moore) and were euthanized 10 days later during luteal phase at 32 weeks of age (April). The ovaries were examined and corpora lutea and large follicles were observed in all postpubertal ewes. Gonadectomies were performed one week prior to euthanizing at 32 weeks. Prior to perfusion, three jugular venous blood samples were collected and LH was measured as described previously (Lee et al., 1976). Body weights and plasma LH levels are shown in Table 1.

**TABLE 1:** Mean (±*SEM*) body weights and plasma LH concentrations of ewes prior to and postpuberty and after GDX

	Prepuberty	Postpuberty	Postpuberty GDX
Bodyweight (kg)	27.25 ± 0.66	28.38 ± 0.80	30.88 ± 0.43
LH (ng/ml)	0.18 ± 0.09	0.33 ± 0.07	1.1 ± 0.41

### 2.2 Tissue processing

The sheep were euthanized with an IV overdose of sodium pentobarbital (Lethabarb; Virbarc) and the brains were perfused with paraformaldehyde as described previously (Smith et al., 2009). Hypothalami were dissected out of the brains and postfixed for 24 hr at 4°C, and then placed in phosphate buffer containing 30% sucrose for 7 days. The hypothalamic blocks were frozen and stored at −20°C. Cryostat sections were cut at 30 µm and stored in cryoprotectant solution at −20°C.

### 2.3 In situ hybridization

Antisense riboprobes for *KISS1*, *TAC3,* *PDYN*, *RFRP,* *GNRH1,* and *KISS1R* were synthesized with SP^6^ or T^7^ transcription kits (Ambion), using corresponding DNA fragments as templates. A 262bp antisense riboprobe for *TAC3* (Genbank accession number XM_004006562.1, bases 31–292) was prepared as described previously (Li, Millar, Clarke, & Smith, 2015). For *PDYN*, a 200 bp fragment of the bovine gene in pCRII (Genbank accession number U58500.1) was provided as a gift by Dr. Hong Jiang, University of Missouri); the use of this probe in sheep has been reported previously (Iqbal, Henry, Pompolo, Rao, & Clarke, 2003). The *KISS1*‐specific template spanned bases 1–357 of the partial ovine cDNA sequence (GenBank accession no. DQ059506) and a 460 bp cDNA sequence of the ovine *RFRP* (bases 43–502, GenBank accession no.NM_001127268) was cloned as previously described (Clarke et al., 2008). The *KISS1R*‐specific template spanned bases 6–636 of ovine cDNA sequence (GenBank accession no. EU272411). The *GNRH1*‐specific template spanned bases 18–169 of the ovine partial cDNA sequence (GenBank accession No. U02517).

In situ hybridization was performed as described previously (Simmons, Arriza, & Swanson, 1989) using ^35^S‐labeled probes. For each sheep and each gene, three sections were selected for analysis. ARC sections were selected to represent the rostral, middle, and caudal regions. The caudal section was 150–300 µm from mammillary recess of the third ventricle and the middle and rostral sections are 600 µm apart. For sections used in *RFRP* detection, rostrocaudal sections of PVN/DMH were chosen: the caudal section was 300–450 µm from mammillary recess of the third ventricle and the middle and rostral sections are 600 µm apart. These were mounted onto SuperFrost plus slides and air‐dried overnight. Following 0.001% proteinase K treatment for 30 min at 37°C, sections were acetylated with 0.0025% acetic anhydride in 0.1 M TEA for 10 min. After rinsing in 2 × SSC, the sections were dehydrated through ascending series of ethanol, delipidated in chloroform, rinsed in absolute ethanol, and air‐dried. The hybridization solutions containing ^35^S labeled antisense probe (5 × 10^6^ cpm/ml) in a cocktail solution of 50% formamide, 5 × SSC, pH 7.0, 250 µg/ml herring sperm DNA, 100 µg/ml yeast tRNA, 5% dextran sulfate, 1× Denhardt's solution, 0.1% Tween‐20 was applied to sections and hybridized overnight at 53°C. After hybridization, sections were washed in decreasing concentrations of SSC, dehydrated, and coated with emulsion (Ilford Imaging). Exposure was 1 week in the dark at 4°C.

Double label in situ hybridization using DIG‐labeled *GNRH1* and ^35^S‐labeled *KISS1R* riboprobes was performed as described previously (Li, Goodchild, Seyedabadi, & Pilowsky, 2005; Li, Rao, Pereira, Clarke, & Smith, 2011; Smith et al., 2011). Rostral, medial, and caudal regions were hybridized with the DIG‐*GnRH1* riboprobe and the ^35^S‐labeled *KISS1R* riboprobes (5 × 10^6^ cpm/ml) at 53°C overnight. After posthybridization washes with descending concentrations of citrate acid and NaCl (SSC), sections were rinsed twice in Tris‐buffered saline (TBS) (0.1 M Tris–HCl, 0.9% NaCl, pH 7.4). The *GNRH1* expressing neurons were revealed with an alkaline phosphatase conjugated goat anti‐digoxigenin antibody (dilution 1:1,000; Roche) and a colorimetric solution of nitro‐blue tetrazolium and 5‐bromo‐4‐chloro‐3‐indolyl phosphate salts (Roche). The ^35^S signal for *KISS1R* was revealed on GnRH neurons, as silver grain staining. The sections were coated with 3% parlodion in isoamyl acetate, dried, dipped in photographic emulsion (Ilford Imaging), and left at 4°C for 5 weeks. Grain‐counting software (Image‐Pro plus; Media Cybernetics) was used to count the number of *KISS1R* mRNA silver grains over each GnRH cell under darkfield illumination. The signal‐to‐noise ratio was set at the 3× background.

For each gene, a sense probe, using the same template as antisense, was used as a negative control to assess nonspecific hybridization.

### 2.4 Microscopy

Image analysis was carried out using randomly coded slides under dark‐field illumination with software designed to count the total number of cells and the number of silver grains per cell (ImagePro plus, Media Cybernetics, Inc.). Cells were counted when silver grain density was three times greater than the background. Data are expressed as the mean number of identifiable cells/section and the mean number of silver grains/cell (a semiquantitative index of mRNA expression/cell). Densitometry (expression/cell) for *PDYN* was not performed as there were very few cells detected in the ARC.

### 2.5 LH Radioimmunoassay

Plasma LH concentrations were measured in duplicate, using the method of Lee et al. (1976). Assay sensitivity was 0.1 ng/ml and the intra‐assay coefficient of variation (CV) was less than 10% over the range of 0.6–15 ng/ml.

### 2.6 Statistical analysis

All grouped data are presented as the means (±*SEM*). Statistical analyses were conducted after checking for heterogeneity of variance, by one‐way ANOVA. Differences were considered significant at *p* < .05.

## 3 RESULTS

Plasma LH levels increased from 0.33 ± 0.07 ng/ml to 1.1 ± 0.41 ng/ml following GDX (*p* < .05) (Table 1).

**FIGURE 1:**
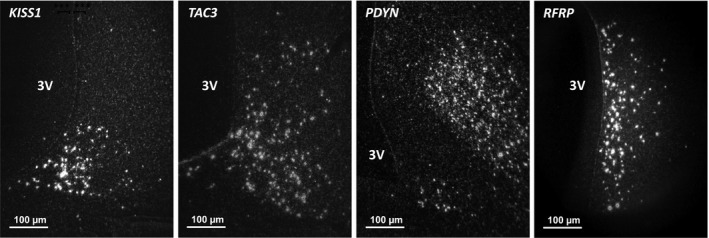
Representative microphotographs of the in situ hybridization signal for *KISS1*, *TAC3*, *PDYN* in arcuate nucleus (ARC) and *RFRP* in the dorsomedial hypothalamus

Examples of the in situ hybridization signal for *KISS1*, *TAC3,* *PDYN*, and *RFRP* are shown in Figure 1. These images indicate high signal‐to‐noise and clear silver grain concentration over the relevant cells. For all probes, no signal was observed after the application of radioactive‐labeled sense probes.

### 3.1 *KISS1*, *TAC3*, *PDYN*, and *RFRP* expression

**FIGURE 2:**
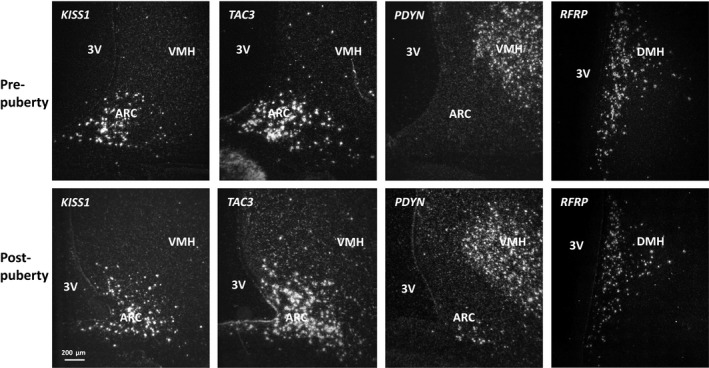
Examples of *KISS1*, *TAC3*, *PDYN* gene expression at the same level in arcuate nucleus and *RFRP* in the dorsalmedial nucleus prior to and after puberty in ewes. Note the fewer number of *PDYN* expressing cells. ARC, arcuate nucleus; VMH, ventromedial hypothalamus; DMH, dorsalmedial hypothalamus; 3V, third ventricle

**FIGURE 3:**
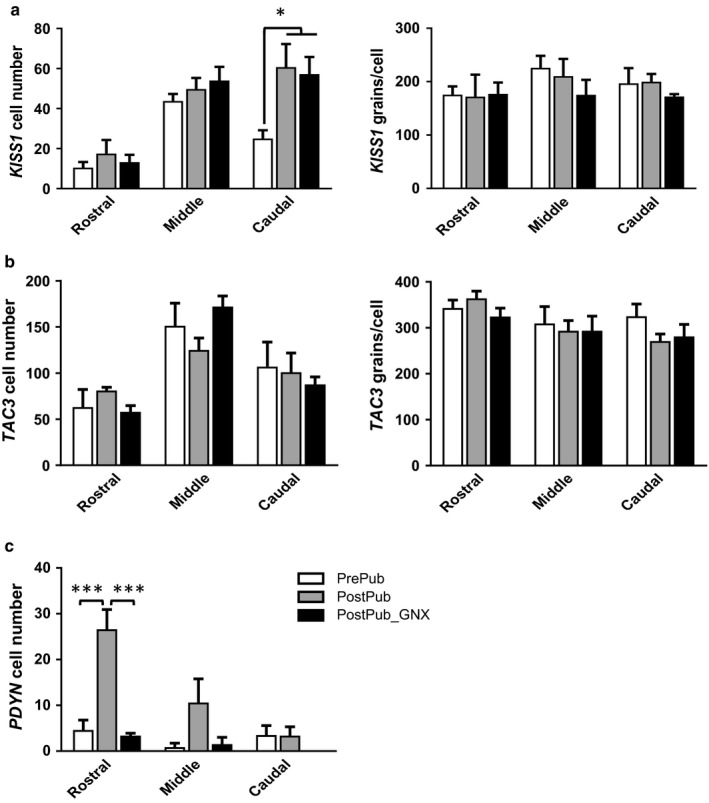
Mean (±*SEM*) expression of *KISS1* (a), *TAC3* (b), and *PDYN* (c) in the ARC of ewes prior to and postpuberty and following GDX. Panels on the left show cell number and those on the right show expression/cell (silver grains/cell) in rostral mid and caudal sections of the ARC. *p < .05, ***p < .001

*KISS1* cell number in the caudal ARC was higher (*p* < .05) in postpubertal ewes than in prepubertal ewes (Figures 2 and 3a), with no change in expression/cell (Figure 3a). GDX did not change *KISS1* expression in postpubertal females (Figure 3a).

The number of TAC3 cells and the expression of *TAC3*/cell was similar in prepubertal, postpubertal intact and GDX ewes (Figures 2 and 3b).

*PDYN* mRNA was highly expressed in supraoptic nucleus (SOR), dorsal medial hypothalamus (DMH) (data not shown), and the ventromedial hypothalamus (Figure 2). *PDYN* expression was low in the ARC, with the number of cells being less than observed for *KISS1* and *TAC3* cells (Figures 2 and 3). *PDYN* cell number increased (*p* < .01) in the rostral ARC across puberty and GDX reduced cell numbers (Figure 3c, *p* < .001).

**FIGURE 4:**
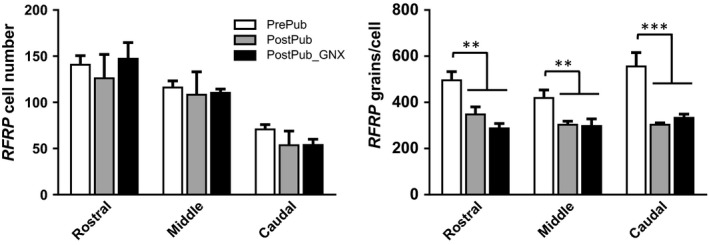
Mean (±*SEM*) *RFRP* gene expression in the DMH of ewes prior to and postpuberty and following GDX. Panels on the left show cell number and those on the right show expression/cell (silver grains/cell) in rostral mid and caudal sections of the dorsomedial nucleus. **p < .01, ***p < .001

The number of *RFRP*‐expressing cells was similar in prepubertal, postpubertal intact, and GDX females, but *RFRP* expression/cell was lower (*p* < .01) after puberty at all levels of the dorsomedial nucleus (Figure 4). The level of expression after puberty was not affected by GDX (Figure 4).

### 3.2 *KISS1R* expression in *GNRH1* cells

**FIGURE 5:**
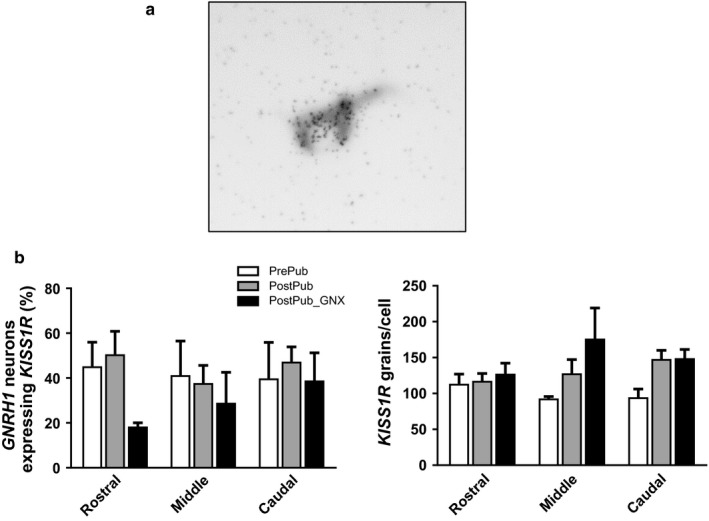
*KISS1R* expression in *GnRH1* cells. Panel A shows co‐localization of *KISS1R* (silver grains; ^35^S‐labeled riboprobe) in a Digoxigenin‐labeled *GNRH1* neuron visualized in gray‐scale. Panel B shows mean (±*SEM*) % *KISSR* expression by *GNRH1* cells (left panel) and silver grain density per cell (right panel)

The number of *GNRH1* expressing neurons was similar in pre and postpubertal sheep (intact or GDX (data not shown). Here ^35^S labeled *KISS1R* expression was distinctively visible on digoxigenin‐labeled GnRH neurons (Figure 5a). *KISS1R* expression/cell and the percentage of GnRH neurons that expressed *KISS1R* were similar before and after puberty (Figure 5b).

## 4 DISCUSSION

It is generally accepted that kisspeptin signaling is mandatory for puberty (d'Anglemont de Tassigny & Colledge, 2010; Dungan Lemko & Elias, 2012; de Roux et al., 2003; Seminara et al., 2003; Topaloglu et al., 2012), but as to whether an increase in expression of *KISS1* or *KISS1R* is seen at this time across all species is questionable. In this study, we focused on the *KISS1* cells of the ARC of the sheep, because an increase in expression.in the POA was recorded in earlier work (Redmond et al., 2011). The present data show that the number of cells expressing *KISS1* in the caudal ARC is higher shortly after puberty in female sheep. The increased number of *KISS1* expression in the ARC of gonad‐intact ewes after puberty is consistent with the increased expression seen in the POA of OVX ewes‐bearing estrogen implants (Redmond et al., 2011). The rise in *KISS1* function in the POA at the time of puberty seems most likely dependent on the action of estrogen on *KISS1* neurons in this location, based on studies in mice (Clarkson, Han, Liu, Lee, & Herbison, 2010). On the other hand, some studies report no change in expression of *KISS1* was seen across the pubertal period in female pigs or in mice and rats of either sex (see Introduction).

We found no change of expression of *KISS1* in the ARC of ewes after GDX soon after puberty, which contrasts with our earlier results obtained with mature ewes (Smith, Clay, Caraty, & Clarke, 2007). This may be because the animals of this study were euthanized one week after GDX or it could be due to there being relatively less influence of gonadal steroids on *KISS1* expression in the early postpubertal stage of development. Certainly there is overwhelming evidence that *KISS1* expression in the ARC increases with GDX of either sex in adult rodents (Irwig et al., 2004; Kauffman et al., 2007; Navarro et al., 2009; Smith, Cunningham, et al., 2005; Smith, Dungan, et al., 2005) and an increase is also seen in female primates after GDX or menopause (Kim, Jessen, Auger, & Terasawa, 2009; Rometo & Rance, 2008). Despite there being no increase in *KISS1* after GDX, the plasma levels of LH increased in both males and females. This suggests that the negative feedback effects of gonadal steroids on GnRH secretion may be mediated by neural elements other than KNDY cells. Certainly, a number of neuronal systems express estrogen receptors (Clarke & Tilbrook, 2009) and could be involved in the suppression of GnRH/LH secretion at this time. In addition, the gonadotropes express sex steroid receptors and negative feedback is effected at that level (Clarke, Cummins, Crowder, & Nett, 1989).

The percentage of *GNRH1* cells expressing *KISS1R* did not change across puberty. The lack of effect of GDX is consistent with results in male rhesus monkeys at the time of expected puberty (Shahab et al., 2005), with similar results in male mice (Molnar et al., 2016) and female rats (Adachi et al., 2007). Neither does *KISS1R* expression change in female rhesus monkeys in the transition to menopause (Kim et al., 2009). On balance, it seems most likely that the transition through puberty is associated with upregulation of kisspeptin activity rather than that of its receptor.

Regarding *TAC3* expression we found no change across puberty or after GDX, consistent with immunohistochemical data on female sheep (Nestor et al., 2012). Our data also concur with data obtained from rats (Navarro et al., 2012). Others (Gill et al., 2012) presented data from female mice and showed that *TAC3* and its receptor (*TAC3R*) were most likely markers of pubertal activation but were not triggers for puberty while data from male mice showed no change in *TAC3R* (Molnar et al., 2016). Overall, there seems to be no significant role for NKB in the initiation of puberty in either sex, even though mutations in *TAC3* and *TAC3R* genes lead to reproductive failure (Silveira et al., 2010).

Around the time of puberty, the number of detectable *PDYN* neurons was lower than for *KISS1* and *TAC3* neurons, which is consistent with other data obtained in female sheep showing very few *PDYN* cells in the prepubertal ewe (Lopez et al., 2016). The striking difference in the number of neurons expressing *TAC3*, *KISS1*, and *PDYN* at this stage of development is interesting, considering that the three peptides co‐localized in the KNDy neurons in the adult sheep (Goodman et al., 2007). This suggests that the neuroendocrine axis governing reproductive function is different in young and adult animals and, as suggested earlier (Lopez et al., 2016), a rise in progesterone levels may be necessary for induction of *PDYN* expression, perhaps explaining why the number of cells increased markedly in the rostral ARC, following puberty. In spite of the low number of cells, we showed that GDX reduced the number of *PDYN* cells. These data are consistent with a fundamental role for this peptide in the negative control of pulsatile GnRH secretion. The low cell number precluded meaningful quantification of the level of expression/cell.

A steady increase in GnIH levels in the hypothalami of male and female rats has been seen across development (Iwasa et al., 2012). Likewise, Poling et al (Poling & Kauffman, 2012) showed a steady increase of GnIH levels in a subpopulation of GnIH neurons in mice of both sexes, whereas Quennell et al (Quennell et al., 2010) showed that *RFRP* expression in the dorsomedial nucleus of male mice peaked at 4 weeks. Nevertheless, this maximal level of expression at 4 weeks was followed by a progressive decline up to 8 weeks, although this was not statistically significant. In another study of mice, a marked increase in *RFRP* cell number was observed at 3 weeks of age in males, with a progressive decline occurring between 7 and 13 weeks of age (Sethi, Tsutsui, & Chaturvedi, 2010). We observed a reduction in the level of expression of RFRP/cell in females after puberty which is not consistent with the aforementioned data obtained in rats and mice but is consistent with there being a release from inhibitory influence on GnRH cells at the time of puberty. When considering the role of GnIH in control of reproduction, it should be noted that this peptide is stimulatory in males but inhibitory in females (Ancel et al., 2017). The lack of effect of ovariectomy in females is similar to results obtained in mice (Iwasa et al., 2012; Quennell et al., 2010).

In conclusion, our data from this study indicate an increase in the level of expression of *KISS1* and a reduction in expression of *RFRP* around the time of puberty in ewes. *PDYN* expression is increased across the pubertal transition with no change in expression of *TAC3*. Because of the prominent role that kisspeptin is thought to play in puberty, we also measured *KISS1R* expression in GnRH cells prior to and after puberty, but there were no significant changes in the percentage of cells expressing the receptor and the expression of mRNA. In addition to the role that the genes investigated in this study play in the pubertal transition, other work strongly suggests that a fundamental switch in expression of transcriptional regulators are the drivers of puberty (Lomniczi et al., 2013). Further studies of such regulators would be warranted in a range of species if a comprehensive understanding of puberty is to be elucidated; these could include neuronal systems involved in brain sensing of bodyweight, which are also involved in regulation of reproduction. Expression of relevant receptors by Kisspeptin neurons and the level of input from modulatory afferents would also be informative. Finally, GDX had little effect on the expression of the genes of interest, suggesting that feedback effects of gonadal steroids at this time are different to those seen in the mature animal.

## CONFLICT OF INTEREST

There is no conflict of interest that could be perceived as prejudicing the impartiality of the research reported.

## References

[phy214399-bib-0001] Adachi, S. , Yamada, S. , Takatsu, Y. , Matsui, H. , Kinoshita, M. , Takase, K. , … Maeda, K. (2007). Involvement of anteroventral periventricular metastin/kisspeptin neurons in estrogen positive feedback action on luteinizing hormone release in female rats. Journal of Reproduction and Development, 53, 367–378. 17213691 10.1262/jrd.18146

[phy214399-bib-0002] Ancel, C. , Inglis, M. A. , & Anderson, G. M. (2017). Central RFRP‐3 stimulates LH secretion in male mice and has cycle stage‐dependent inhibitory effects in females. Endocrinology, 158, 2873–2883. 28475692 10.1210/en.2016-1902

[phy214399-bib-0003] Bentsen, A. H. , Ansel, L. , Simonneaux, V. , Tena‐Sempere, M. , Juul, A. , & Mikkelsen, J. D. (2010). Maturation of kisspeptinergic neurons coincides with puberty onset in male rats. Peptides, 31, 275–283. 19944729 10.1016/j.peptides.2009.11.017

[phy214399-bib-0004] Clarke, I. J. (2015). Hypothalamus as an endocrine organ. Comprehensive Physiology, 5, 217–253. 25589270 10.1002/cphy.c140019

[phy214399-bib-0005] Clarke, I. J. , & Arbabi, L. (2015). New concepts of the central control of reproduction, integrating influence of stress, metabolic state and season. Domestic Animal Endocrinology, 56, S165–S179. 10.1016/j.domaniend.2016.03.001 27345314

[phy214399-bib-0006] Clarke, I. J. , Campbell, R. , Smith, J. T. , Prevot, V. , & Wray, S. (2011). Neuroendocrine control of reproduction In Fink G., Pfaff D., & Levine J. (Eds.), Handbook of neuroendocrinology (pp. 198–235). London, UK: Elsevier.

[phy214399-bib-0007] Clarke, I. J. , Cummins, J. T. , Crowder, M. E. , & Nett, T. M. (1989). Long‐term negative feedback effects of oestrogen and progesterone on the pituitary gland of the long‐term ovariectomized ewe. Journal of Endocrinology, 120, 207–214. 2538532 10.1677/joe.0.1200207

[phy214399-bib-0008] Clarke, I. J. , & Parkington, H. C. (2014). Gonadotropin inhibitory hormone (GnIH) as a regulator of gonadotropes. Molecular and Cellular Endocrinology, 385, 36–44. 23994028 10.1016/j.mce.2013.08.017

[phy214399-bib-0009] Clarke, I. J. , & Pompolo, S. (2005). Synthesis and secretion of GnRH. Animal Reproduction Science, 88, 29–55. 15993013 10.1016/j.anireprosci.2005.05.003

[phy214399-bib-0010] Clarke, I. J. , Sari, I. P. , Qi, Y. , Smith, J. T. , Parkington, H. C. , Ubuka, T. , … Bentley, G. E. (2008). Potent action of RFamide‐related peptide‐3 on pituitary gonadotropes indicative of a hypophysiotropic role in the negative regulation of gonadotropin secretion. Endocrinology, 149, 5811–5821. 18617613 10.1210/en.2008-0575

[phy214399-bib-0011] Clarke, I. J. , & Smith, J. T. (2010). The role of kisspeptin and gonadotropin inhibitory hormone (GnIH) in the seasonality of reproduction in sheep. Society for Reproduction and Fertility Supplement, 67, 159–169. 21755670

[phy214399-bib-0012] Clarke, I. J. , Smith, J. T. , Henry, B. A. , Oldfield, B. J. , Stefanidis, A. , Millar, R. P. , … Fraley, G. S. (2012). Gonadotropin‐inhibitory hormone is a hypothalamic peptide that provides a molecular switch between reproduction and feeding. Neuroendocrinology, 95, 305–316. 22286004 10.1159/000332822

[phy214399-bib-0013] Clarke, I. J. , & Tilbrook, A. J. (2009). Gonadotropin, neural and hormonal control. Oxford: Academic Press, Elsevier.

[phy214399-bib-0014] Clarkson, J. , Han, S. K. , Liu, X. , Lee, K. , & Herbison, A. E. (2010). Neurobiological mechanisms underlying kisspeptin activation of gonadotropin‐releasing hormone (GnRH) neurons at puberty. Molecular and Cellular Endocrinology, 324, 45–50. 20109523 10.1016/j.mce.2010.01.026

[phy214399-bib-0015] d'Anglemont de Tassigny, X. , Colledge, W. H. (2010). The role of kisspeptin signaling in reproduction. Physiology, 25, 207–217. 20699467 10.1152/physiol.00009.2010

[phy214399-bib-0016] de Roux, N. , Genin, E. , Carel, J. C. , Matsuda, F. , Chaussain, J. L. , & Milgrom, E. (2003). Hypogonadotropic hypogonadism due to loss of function of the KiSS1‐derived peptide receptor GPR54. Proceedings of the National Academy of Sciences of the United States of America, 100, 10972–10976. 12944565 10.1073/pnas.1834399100 PMC196911

[phy214399-bib-0017] Dungan Lemko, H. M. , & Elias, C. F. (2012). Kiss of the mutant mouse: How genetically altered mice advanced our understanding of kisspeptin's role in reproductive physiology. Endocrinology, 153, 5119–5129. 23011921 10.1210/en.2012-1494 PMC3473196

[phy214399-bib-0018] Eghlidi, D. H. , Haley, G. E. , Noriega, N. C. , Kohama, S. G. , & Urbanski, H. F. (2010). Influence of age and 17beta‐estradiol on kisspeptin, neurokinin B, and prodynorphin gene expression in the arcuate‐median eminence of female rhesus macaques. Endocrinology, 151, 3783–3794. 20519367 10.1210/en.2010-0198 PMC2940528

[phy214399-bib-0019] Estrada, K. M. , Clay, C. M. , Pompolo, S. , Smith, J. T. , & Clarke, I. J. (2006). Elevated KiSS‐1 expression in the arcuate nucleus prior to the cyclic preovulatory gonadotrophin‐releasing hormone/lutenising hormone surge in the ewe suggests a stimulatory role for kisspeptin in oestrogen‐positive feedback. Journal of Neuroendocrinology, 18, 806–809. 16965299 10.1111/j.1365-2826.2006.01485.x

[phy214399-bib-0020] Foradori, C. D. , Goodman, R. L. , Adams, V. L. , Valent, M. , & Lehman, M. N. (2005). Progesterone increases dynorphin a concentrations in cerebrospinal fluid and preprodynorphin messenger ribonucleic acid levels in a subset of dynorphin neurons in the sheep. Endocrinology, 146, 1835–1842. 15650077 10.1210/en.2004-1326

[phy214399-bib-0021] Foster, D. L. , & Ryan, K. D. (1979). Endocrine mechanisms governing transition into adulthood: A marked decrease in inhibitory feedback action of estradiol on tonic secretion of luteinizing hormone in the lamb during puberty. Endocrinology, 105, 896–904. 477603 10.1210/endo-105-4-896

[phy214399-bib-0022] Franceschini, I. , Lomet, D. , Cateau, M. , Delsol, G. , Tillet, Y. , & Caraty, A. (2006). Kisspeptin immunoreactive cells of the ovine preoptic area and arcuate nucleus co‐express estrogen receptor alpha. Neuroscience Letters, 401, 225–230. 16621281 10.1016/j.neulet.2006.03.039

[phy214399-bib-0023] Garcia, J. P. , Keen, K. L. , Kenealy, B. P. , Seminara, S. B. , & Terasawa, E. (2018). Role of kisspeptin and neurokinin B signaling in male rhesus monkey puberty. Endocrinology, 159, 3048–3060. 29982393 10.1210/en.2018-00443 PMC6456982

[phy214399-bib-0024] Gianetti, E. , Tusset, C. , Noel, S. D. , Au, M. G. , Dwyer, A. A. , Hughes, V. A. , … Seminara, S. B. (2010). TAC3/TACR3 mutations reveal preferential activation of gonadotropin‐releasing hormone release by neurokinin B in neonatal life followed by reversal in adulthood. Journal of Clinical Endocrinology and Metabolism, 95, 2857–2867. 20332248 10.1210/jc.2009-2320 PMC2902066

[phy214399-bib-0025] Gibson, E. M. , Humber, S. A. , Jain, S. , Williams III, W. P. , Zhao, S. , Bentley, G. E. , … Kriegsfeld, L. J. (2008). Alterations in RFamide‐related peptide expression are coordinated with the preovulatory luteinizing hormone surge. Endocrinology, 149, 4958–4969. 18566114 10.1210/en.2008-0316 PMC2582915

[phy214399-bib-0026] Gill, J. C. , Navarro, V. M. , Kwong, C. , Noel, S. D. , Martin, C. , Xu, S. , … Kaiser, U. B. (2012). Increased neurokinin B (Tac2) expression in the mouse arcuate nucleus is an early marker of pubertal onset with differential sensitivity to sex steroid‐negative feedback than Kiss1. Endocrinology, 153, 4883–4893. 22893725 10.1210/en.2012-1529 PMC3512019

[phy214399-bib-0027] Gill, J. C. , Wang, O. , Kakar, S. , Martinelli, E. , Carroll, R. S. , & Kaiser, U. B. (2010). Reproductive hormone‐dependent and ‐independent contributions to developmental changes in kisspeptin in GnRH‐deficient hypogonadal mice. PLoS ONE, 5, e11911. 20689830 10.1371/journal.pone.0011911 PMC2912854

[phy214399-bib-0028] Goodman, R. L. , Coolen, L. M. , Anderson, G. M. , Hardy, S. L. , Valent, M. , Connors, J. M. , … Lehman, M. N. (2004). Evidence that dynorphin plays a major role in mediating progesterone negative feedback on gonadotropin‐releasing hormone neurons in sheep. Endocrinology, 145, 2959–2967. 14988383 10.1210/en.2003-1305

[phy214399-bib-0029] Goodman, R. L. , Coolen, L. M. , & Lehman, M. N. (2014). A role for neurokinin B in pulsatile GnRH secretion in the ewe. Neuroendocrinology, 99, 18–32. 24008670 10.1159/000355285 PMC3976461

[phy214399-bib-0030] Goodman, R. L. , Holaskova, I. , Nestor, C. C. , Connors, J. M. , Billings, H. J. , Valent, M. , … Hileman, S. M. (2011). Evidence that the arcuate nucleus is an important site of progesterone negative feedback in the ewe. Endocrinology, 152, 3451–3460. 21693677 10.1210/en.2011-0195 PMC3159787

[phy214399-bib-0031] Goodman, R. L. , Lehman, M. N. , Smith, J. T. , Coolen, L. M. , de Oliveira, C. V. , Jafarzadehshirazi, M. R. , … Clarke, I. J. (2007). Kisspeptin neurons in the arcuate nucleus of the ewe express both dynorphin A and neurokinin B. Endocrinology, 148, 5752–5760. 17823266 10.1210/en.2007-0961

[phy214399-bib-0032] Goodman, R. L. , Parfitt, D. B. , Evans, N. P. , Dahl, G. E. , & Karsch, F. J. (1995). Endogenous opioid peptides control the amplitude and shape of gonadotropin‐releasing hormone pulses in the ewe. Endocrinology, 136, 2412–2420. 7750462 10.1210/endo.136.6.7750462

[phy214399-bib-0033] Gottsch, M. L. , Cunningham, M. J. , Smith, J. T. , Popa, S. M. , Acohido, B. V. , Crowley, W. F. , … Steiner, R. A. (2004). A role for kisspeptins in the regulation of gonadotropin secretion in the mouse. Endocrinology, 145, 4073–4077. 15217982 10.1210/en.2004-0431

[phy214399-bib-0034] Grachev, P. , Li, X. F. , Hu, M. H. , Li, S. Y. , Millar, R. P. , Lightman, S. L. , & O'Byrne, K. T. (2014). Neurokinin B signaling in the female rat: A novel link between stress and reproduction. Endocrinology, 155, 2589–2601. 24708241 10.1210/en.2013-2038

[phy214399-bib-0035] Han, S. K. , Gottsch, M. L. , Lee, K. J. , Popa, S. M. , Smith, J. T. , Jakawich, S. K. , … Herbison, A. E. (2005). Activation of gonadotropin‐releasing hormone neurons by kisspeptin as a neuroendocrine switch for the onset of puberty. Journal of Neuroscience, 25, 11349–11356. 16339030 10.1523/JNEUROSCI.3328-05.2005 PMC6725899

[phy214399-bib-0036] Hoffman, G. E. , Le, W. W. , Franceschini, I. , Caraty, A. , & Advis, J. P. (2011). Expression of fos and in vivo median eminence release of LHRH identifies an active role for preoptic area kisspeptin neurons in synchronized surges of LH and LHRH in the ewe. Endocrinology, 152, 214–222. 21047947 10.1210/en.2010-0066 PMC3219045

[phy214399-bib-0037] I'Anson, H. , Manning, J. M. , Herbosa, C. G. , Pelt, J. , Friedman, C. R. , Wood, R. I. , … Foster, D. L. (2000). Central inhibition of gonadotropin‐releasing hormone secretion in the growth‐restricted hypogonadotropic female sheep. Endocrinology, 141, 520–527. 10650931 10.1210/endo.141.2.7308

[phy214399-bib-0038] Ieda, N. , Uenoyama, Y. , Tajima, Y. , Nakata, T. , Kano, M. , Naniwa, Y. , … Tsukamura, H. (2014). KISS1 gene expression in the developing brain of female pigs in pre‐ and peripubertal periods. Journal of Reproduction and Development, 60, 312–316. 24909600 10.1262/jrd.2013-129 PMC4139506

[phy214399-bib-0039] Iqbal, J. , Henry, B. A. , Pompolo, S. , Rao, A. , & Clarke, I. J. (2003). Long‐term alteration in bodyweight and food restriction does not affect the gene expression of either preproorexin or prodynorphin in the sheep. Neuroscience, 118, 217–226. 12676151 10.1016/s0306-4522(02)00815-1

[phy214399-bib-0040] Irwig, M. S. , Fraley, G. S. , Smith, J. T. , Acohido, B. V. , Popa, S. M. , Cunningham, M. J. , … Steiner, R. A. (2004). Kisspeptin activation of gonadotropin releasing hormone neurons and regulation of KiSS‐1 mRNA in the male rat. Neuroendocrinology, 80, 264–272. 15665556 10.1159/000083140

[phy214399-bib-0041] Iwasa, T. , Matsuzaki, T. , Murakami, M. , Kinouchi, R. , Osugi, T. , Gereltsetseg, G. , … Tsutsui, K. (2012). Developmental changes in the mammalian gonadotropin‐inhibitory hormone (GnIH) ortholog RFamide‐related peptide (RFRP) and its cognate receptor GPR147 in the rat hypothalamus. International Journal of Developmental Neuroscience, 30, 31–37. 22064075 10.1016/j.ijdevneu.2011.10.003

[phy214399-bib-0042] Johnson, M. A. , & Fraley, G. S. (2008). Rat RFRP‐3 alters hypothalamic GHRH expression and growth hormone secretion but does not affect KiSS‐1 gene expression or the onset of puberty in male rats. Neuroendocrinology, 88, 305–315. 18628614 10.1159/000145718

[phy214399-bib-0043] Johnson, M. A. , Tsutsui, K. , & Fraley, G. S. (2007). Rat RFamide‐related peptide‐3 stimulates GH secretion, inhibits LH secretion, and has variable effects on sex behavior in the adult male rat. Hormones and Behavior, 51, 171–180. 17113584 10.1016/j.yhbeh.2006.09.009 PMC1831848

[phy214399-bib-0044] Kadokawa, H. , Shibata, M. , Tanaka, Y. , Kojima, T. , Matsumoto, K. , Oshima, K. , & Yamamoto, N. (2009). Bovine C‐terminal octapeptide of RFamide‐related peptide‐3 suppresses luteinizing hormone (LH) secretion from the pituitary as well as pulsatile LH secretion in bovines. Domestic Animal Endocrinology, 36, 219–224. 19328642 10.1016/j.domaniend.2009.02.001

[phy214399-bib-0045] Kauffman, A. S. , Gottsch, M. L. , Roa, J. , Byquist, A. C. , Crown, A. , Clifton, D. K. , … Tena‐Sempere, M. (2007). Sexual differentiation of Kiss1 gene expression in the brain of the rat. Endocrinology, 148, 1774–1783. 17204549 10.1210/en.2006-1540

[phy214399-bib-0046] Kim, W. , Jessen, H. M. , Auger, A. P. , & Terasawa, E. (2009). Postmenopausal increase in KiSS‐1, GPR54, and luteinizing hormone releasing hormone (LHRH‐1) mRNA in the basal hypothalamus of female rhesus monkeys. Peptides, 30, 103–110. 18619506 10.1016/j.peptides.2008.06.005 PMC2612733

[phy214399-bib-0047] Kriegsfeld, L. J. , Mei, D. F. , Bentley, G. E. , Ubuka, T. , Mason, A. O. , Inoue, K. , … Silver, R. (2006). Identification and characterization of a gonadotropin‐inhibitory system in the brains of mammals. Proceedings of the National Academy of Sciences of the United States of America, 103, 2410–2415. 16467147 10.1073/pnas.0511003103 PMC1413747

[phy214399-bib-0048] Lee, V. W. , Cumming, I. A. , de Kretser, D. M. , Findlay, J. K. , Hudson, B. , & Keogh, E. J. (1976). Regulation of gonadotrophin secretion in rams from birth to sexual maturity. I. Plasma LH, FSH and testosterone levels. Journal of Reproduction and Fertility, 46, 1–6. 1271330 10.1530/jrf.0.0460001

[phy214399-bib-0049] Lehman, M. N. , Coolen, L. M. , & Goodman, R. L. (2010). Minireview: Kisspeptin/neurokinin B/dynorphin (KNDy) cells of the arcuate nucleus: A central node in the control of gonadotropin‐releasing hormone secretion. Endocrinology, 151, 3479–3489. 20501670 10.1210/en.2010-0022 PMC2940527

[phy214399-bib-0050] Leon, S. , Garcia‐Galiano, D. , Ruiz‐Pino, F. , Barroso, A. , Manfredi‐Lozano, M. , Romero‐Ruiz, A. , … Tena‐Sempere, M. (2014). Physiological roles of gonadotropin‐inhibitory hormone signaling in the control of mammalian reproductive axis: Studies in the NPFF1 receptor null mouse. Endocrinology, 155, 2953–2965. 24823392 10.1210/en.2014-1030

[phy214399-bib-0051] Li, Q. , Goodchild, A. K. , Seyedabadi, M. , & Pilowsky, P. M. (2005). Pre‐protachykinin A mRNA is colocalized with tyrosine hydroxylase‐immunoreactivity in bulbospinal neurons. Neuroscience, 136, 205–216. 16198496 10.1016/j.neuroscience.2005.07.057

[phy214399-bib-0052] Li, Q. , Millar, R. P. , Clarke, I. J. , & Smith, J. T. (2015). Evidence that neurokinin B controls basal gonadotropin‐releasing hormone secretion but is not critical for estrogen‐positive feedback in sheep. Neuroendocrinology, 101, 161–174. 25677216 10.1159/000377702

[phy214399-bib-0053] Li, Q. , Rao, A. , Pereira, A. , Clarke, I. J. , & Smith, J. T. (2011). Kisspeptin cells in the ovine arcuate nucleus express prolactin receptor but not melatonin receptor. Journal of Neuroendocrinology, 23, 871–882. 21793946 10.1111/j.1365-2826.2011.02195.x

[phy214399-bib-0054] Lomniczi, A. , Loche, A. , Castellano, J. M. , Ronnekleiv, O. K. , Bosch, M. , Kaidar, G. , … Ojeda, S. R. (2013). Epigenetic control of female puberty. Nature Neuroscience, 16, 281–289. 23354331 10.1038/nn.3319 PMC3581714

[phy214399-bib-0055] Lopez, J. A. , Bedenbaugh, M. N. , McCosh, R. B. , Weems, P. W. , Meadows, L. J. , Wisman, B. , … Hileman, S. M. (2016). Does dynorphin play a role in the onset of puberty in female sheep? Journal of Neuroendocrinology, 28 10.1111/jne.12445 PMC5412962 28328155

[phy214399-bib-0056] Maeda, K. , Ohkura, S. , Uenoyama, Y. , Wakabayashi, Y. , Oka, Y. , Tsukamura, H. , & Okamura, H. (2010). Neurobiological mechanisms underlying GnRH pulse generation by the hypothalamus. Brain Research, 1364, 103–115. 20951683 10.1016/j.brainres.2010.10.026

[phy214399-bib-0057] Messager, S. , Chatzidaki, E. E. , Ma, D. , Hendrick, A. G. , Zahn, D. , Dixon, J. , … Aparicio, S. A. (2005). Kisspeptin directly stimulates gonadotropin‐releasing hormone release via G protein‐coupled receptor 54. Proceedings of the National Academy of Sciences of the United States of America, 102, 1761–1766. 15665093 10.1073/pnas.0409330102 PMC545088

[phy214399-bib-0058] Molnar, C. S. , Sarvari, M. , Vastagh, C. , Maurnyi, C. , Fekete, C. , Liposits, Z. , & Hrabovszky, E. (2016). Altered gene expression profiles of the hypothalamic arcuate nucleus of male mice suggest profound developmental changes in peptidergic signaling. Neuroendocrinology, 103, 369–382. 26338351 10.1159/000439430

[phy214399-bib-0059] Murakami, M. , Matsuzaki, T. , Iwasa, T. , Yasui, T. , Irahara, M. , Osugi, T. , & Tsutsui, K. (2008). Hypophysiotropic role of RFamide‐related peptide‐3 in the inhibition of LH secretion in female rats. Journal of Endocrinology, 199, 105–112. 18653621 10.1677/JOE-08-0197

[phy214399-bib-0060] Nakahara, T. , Uenoyama, Y. , Iwase, A. , Oishi, S. , Nakamura, S. , Minabe, S. , … Tsukamura, H. (2013). Chronic peripheral administration of kappa‐opioid receptor antagonist advances puberty onset associated with acceleration of pulsatile luteinizing hormone secretion in female rats. Journal of Reproduction and Development, 59, 479–484. 23877505 10.1262/jrd.2013-046 PMC3934117

[phy214399-bib-0061] Navarro, V. M. , Gottsch, M. L. , Chavkin, C. , Okamura, H. , Clifton, D. K. , & Steiner, R. A. (2009). Regulation of gonadotropin‐releasing hormone secretion by kisspeptin/dynorphin/neurokinin B neurons in the arcuate nucleus of the mouse. Journal of Neuroscience, 29, 11859–11866. 19776272 10.1523/JNEUROSCI.1569-09.2009 PMC2793332

[phy214399-bib-0062] Navarro, V. M. , Ruiz‐Pino, F. , Sanchez‐Garrido, M. A. , Garcia‐Galiano, D. , Hobbs, S. J. , Manfredi‐Lozano, M. , … Tena‐Sempere, M. (2012). Role of neurokinin B in the control of female puberty and its modulation by metabolic status. Journal of Neuroscience, 32, 2388–2397. 22396413 10.1523/JNEUROSCI.4288-11.2012 PMC3567461

[phy214399-bib-0063] Nestor, C. C. , Briscoe, A. M. , Davis, S. M. , Valent, M. , Goodman, R. L. , & Hileman, S. M. (2012). Evidence of a role for kisspeptin and neurokinin B in puberty of female sheep. Endocrinology, 153, 2756–2765. 22434087 10.1210/en.2011-2009 PMC3359609

[phy214399-bib-0064] Ojeda, S. R. , Lomniczi, A. , Mastronardi, C. , Heger, S. , Roth, C. , Parent, A. S. , … Mungenast, A. E. (2006). Minireview: The neuroendocrine regulation of puberty: Is the time ripe for a systems biology approach? Endocrinology, 147, 1166–1174. 16373420 10.1210/en.2005-1136

[phy214399-bib-0065] Ojeda, S. R. , Roth, C. , Mungenast, A. , Heger, S. , Mastronardi, C. , Parent, A. S. , … Jung, H. (2006). Neuroendocrine mechanisms controlling female puberty: New approaches, new concepts. International Journal of Andrology, 29, 256–263; discussion 286–90. 16466547 10.1111/j.1365-2605.2005.00619.x

[phy214399-bib-0066] Plant, T. M. (2015). Neuroendocrine control of the onset of puberty. Frontiers in Neuroendocrinology, 38, 73–88. 25913220 10.1016/j.yfrne.2015.04.002 PMC4457677

[phy214399-bib-0067] Plant, T. M. , & Shahab, M. (2002). Neuroendocrine mechanisms that delay and initiate puberty in higher primates. Physiology & Behavior, 77, 717–722. 12527025 10.1016/s0031-9384(02)00924-1

[phy214399-bib-0068] Poling, M. C. , & Kauffman, A. S. (2012). Sexually dimorphic testosterone secretion in prenatal and neonatal mice is independent of kisspeptin‐Kiss1r and GnRH signaling. Endocrinology, 153, 782–793. 22202164 10.1210/en.2011-1838 PMC3275395

[phy214399-bib-0069] Poling, M. C. , Kim, J. , Dhamija, S. , & Kauffman, A. S. (2012). Development, sex steroid regulation, and phenotypic characterization of RFamide‐related peptide (Rfrp) gene expression and RFamide receptors in the mouse hypothalamus. Endocrinology, 153, 1827–1840. 22355072 10.1210/en.2011-2049 PMC3320244

[phy214399-bib-0070] Quennell, J. H. , Rizwan, M. Z. , Relf, H. L. , & Anderson, G. M. (2010). Developmental and steroidogenic effects on the gene expression of RFamide related peptides and their receptor in the rat brain and pituitary gland. Journal of Neuroendocrinology, 22, 309–316. 20136694 10.1111/j.1365-2826.2010.01963.x

[phy214399-bib-0071] Ramaswamy, S. , Seminara, S. B. , Ali, B. , Ciofi, P. , Amin, N. A. , & Plant, T. M. (2010). Neurokinin B stimulates GnRH release in the male monkey (*Macaca mulatta*) and is colocalized with kisspeptin in the arcuate nucleus. Endocrinology, 151, 4494–4503. 20573725 10.1210/en.2010-0223 PMC2940495

[phy214399-bib-0072] Rance, N. E. , Krajewski, S. J. , Smith, M. A. , Cholanian, M. , & Dacks, P. A. (2010). Neurokinin B and the hypothalamic regulation of reproduction. Brain Research, 1364, 116–128. 20800582 10.1016/j.brainres.2010.08.059 PMC2992576

[phy214399-bib-0073] Redmond, J. S. , Baez‐Sandoval, G. M. , Spell, K. M. , Spencer, T. E. , Lents, C. A. , Williams, G. L. , & Amstalden, M. (2011). Developmental changes in hypothalamic Kiss1 expression during activation of the pulsatile release of luteinising hormone in maturing ewe lambs. Journal of Neuroendocrinology, 23, 815–822. 21679258 10.1111/j.1365-2826.2011.02177.x

[phy214399-bib-0074] Robertson, J. L. , Clifton, D. K. , de la Iglesia, H. O. , Steiner, R. A. , & Kauffman, A. S. (2009). Circadian regulation of Kiss1 neurons: Implications for timing the preovulatory gonadotropin‐releasing hormone/luteinizing hormone surge. Endocrinology, 150, 3664–3671. 19443569 10.1210/en.2009-0247 PMC2717859

[phy214399-bib-0075] Rometo, A. M. , & Rance, N. E. (2008). Changes in prodynorphin gene expression and neuronal morphology in the hypothalamus of postmenopausal women. Journal of Neuroendocrinology, 20, 1376–1381. 19094085 10.1111/j.1365-2826.2008.01796.x PMC2893873

[phy214399-bib-0076] Ruiz‐Pino, F. , Navarro, V. M. , Bentsen, A. H. , Garcia‐Galiano, D. , Sanchez‐Garrido, M. A. , Ciofi, P. , … Tena‐Sempere, M. (2012). Neurokinin B and the control of the gonadotropic axis in the rat: Developmental changes, sexual dimorphism, and regulation by gonadal steroids. Endocrinology, 153, 4818–4829. 22822161 10.1210/en.2012-1287 PMC3512006

[phy214399-bib-0077] Sakamoto, K. , Murata, K. , Wakabayashi, Y. , Yayou, K. , Ohkura, S. , Takeuchi, Y. , … Okamura, H. (2012). Central administration of neurokinin B activates kisspeptin/NKB neurons in the arcuate nucleus and stimulates luteinizing hormone secretion in ewes during the non‐breeding season. Journal of Reproduction and Development, 58, 700–706. 22972185 10.1262/jrd.2011-038

[phy214399-bib-0078] Seminara, S. B. , Messager, S. , Chatzidaki, E. E. , Thresher, R. R. , Acierno Jr, J. S. , Shagoury, J. K. , … Colledge Jr, W. H. (2003). The GPR54 gene as a regulator of puberty. New England Journal of Medicine, 349, 1614–1627. 14573733 10.1056/NEJMoa035322

[phy214399-bib-0079] Sethi, S. , Tsutsui, K. , & Chaturvedi, C. M. (2010). Age‐dependent variation in the RFRP‐3 neurons is inversely correlated with gonadal activity of mice. General and Comparative Endocrinology, 168, 326–332. 20433842 10.1016/j.ygcen.2010.04.011

[phy214399-bib-0080] Shahab, M. , Mastronardi, C. , Seminara, S. B. , Crowley, W. F. , Ojeda, S. R. , & Plant, T. M. (2005). Increased hypothalamic GPR54 signaling: A potential mechanism for initiation of puberty in primates. Proceedings of the National Academy of Sciences of the United States of America, 102, 2129–2134. 15684075 10.1073/pnas.0409822102 PMC548549

[phy214399-bib-0081] Silveira, L. F. , Trarbach, E. B. , & Latronico, A. C. (2010). Genetics basis for GnRH‐dependent pubertal disorders in humans. Molecular and Cellular Endocrinology, 324, 30–38. 20188792 10.1016/j.mce.2010.02.023

[phy214399-bib-0082] Simmons, D. M. , Arriza, J. L. , & Swanson, L. W. (1989). A complete protocol for in situ hybridization of messager RNA in brain and other tissue with radiolabeled single‐stranded RNA probes. Journal of Histotechnology, 12, 169–181.

[phy214399-bib-0083] Smith, J. T. (2008). Kisspeptin signalling in the brain: Steroid regulation in the rodent and ewe. Brain Research Reviews, 57, 288–298. 17509691 10.1016/j.brainresrev.2007.04.002

[phy214399-bib-0084] Smith, J. T. , Clay, C. M. , Caraty, A. , & Clarke, I. J. (2007). KiSS‐1 messenger ribonucleic acid expression in the hypothalamus of the ewe is regulated by sex steroids and season. Endocrinology, 148, 1150–1157. 17185374 10.1210/en.2006-1435

[phy214399-bib-0085] Smith, J. T. , Cunningham, M. J. , Rissman, E. F. , Clifton, D. K. , & Steiner, R. A. (2005). Regulation of Kiss1 gene expression in the brain of the female mouse. Endocrinology, 146, 3686–3692. 15919741 10.1210/en.2005-0488

[phy214399-bib-0086] Smith, J. T. , Dungan, H. M. , Stoll, E. A. , Gottsch, M. L. , Braun, R. E. , Eacker, S. M. , … Steiner, R. A. (2005). Differential regulation of KiSS‐1 mRNA expression by sex steroids in the brain of the male mouse. Endocrinology, 146, 2976–2984. 15831567 10.1210/en.2005-0323

[phy214399-bib-0087] Smith, J. T. , Li, Q. , Pereira, A. , & Clarke, I. J. (2009). Kisspeptin neurons in the ovine arcuate nucleus and preoptic area are involved in the preovulatory luteinizing hormone surge. Endocrinology, 150, 5530–5538. 19819940 10.1210/en.2009-0712

[phy214399-bib-0088] Smith, J. T. , Li, Q. , Yap, K. S. , Shahab, M. , Roseweir, A. K. , Millar, R. P. , & Clarke, I. J. (2011). Kisspeptin is essential for the full preovulatory LH surge and stimulates GnRH release from the isolated ovine median eminence. Endocrinology, 152, 1001–1012. 21239443 10.1210/en.2010-1225

[phy214399-bib-0089] Smith, J. T. , Popa, S. M. , Clifton, D. K. , Hoffman, G. E. , & Steiner, R. A. (2006). Kiss1 neurons in the forebrain as central processors for generating the preovulatory luteinizing hormone surge. Journal of Neuroscience, 26, 6687–6694. 16793876 10.1523/JNEUROSCI.1618-06.2006 PMC6673844

[phy214399-bib-0090] Takase, K. , Uenoyama, Y. , Inoue, N. , Matsui, H. , Yamada, S. , Shimizu, M. , … Maeda, K. I. (2009). Possible role of oestrogen in pubertal increase of Kiss1/kisspeptin expression in discrete hypothalamic areas of female rats. Journal of Neuroendocrinology, 21, 527–537. 19500223 10.1111/j.1365-2826.2009.01868.x

[phy214399-bib-0091] Tena‐Sempere, M. (2012). Deciphering puberty: Novel partners, novel mechanisms. European Journal of Endocrinology, 167, 733–747. 22989465 10.1530/EJE-12-0669

[phy214399-bib-0092] Terasawa, E. , & Fernandez, D. L. (2001). Neurobiological mechanisms of the onset of puberty in primates. Endocrine Reviews, 22, 111–151. 11159818 10.1210/edrv.22.1.0418

[phy214399-bib-0093] Topaloglu, A. K. , Reimann, F. , Guclu, M. , Yalin, A. S. , Kotan, L. D. , Porter, K. M. , … Semple, R. K. (2009). TAC3 and TACR3 mutations in familial hypogonadotropic hypogonadism reveal a key role for Neurokinin B in the central control of reproduction. Nature Genetics, 41, 354–358. 19079066 10.1038/ng.306 PMC4312696

[phy214399-bib-0094] Topaloglu, A. K. , Tello, J. A. , Kotan, L. D. , Ozbek, M. N. , Yilmaz, M. B. , Erdogan, S. , … Yuksel, B. (2012). Inactivating KISS1 mutation and hypogonadotropic hypogonadism. New England Journal of Medicine, 366, 629–635. 22335740 10.1056/NEJMoa1111184

[phy214399-bib-0095] Tsutsui, K. , Saigoh, E. , Ukena, K. , Teranishi, H. , Fujisawa, Y. , Kikuchi, M. , … Sharp, P. J. (2000). A novel avian hypothalamic peptide inhibiting gonadotropin release. Biochemical and Biophysical Research Communications, 275, 661–667. 10964719 10.1006/bbrc.2000.3350

[phy214399-bib-0096] Weems, P. W. , Witty, C. F. , Amstalden, M. , Coolen, L. M. , Goodman, R. L. , & Lehman, M. N. (2016). kappa‐Opioid receptor is colocalized in GnRH and KNDy cells in the female ovine and rat brain. Endocrinology, 157, 2367–2379. 27064940 10.1210/en.2015-1763 PMC4891780

[phy214399-bib-0097] Yen, S. S. , Quigley, M. E. , Reid, R. L. , Ropert, J. F. , & Cetel, N. S. (1985). Neuroendocrinology of opioid peptides and their role in the control of gonadotropin and prolactin secretion. American Journal of Obstetrics and Gynecology, 152, 485–493. 2990210 10.1016/s0002-9378(85)80162-9

